# Breadth and depth outreach of Islamic cooperatives: do size, non-performing finance, and grant matter?

**DOI:** 10.1016/j.heliyon.2020.e04472

**Published:** 2020-07-27

**Authors:** Wasiaturrahma, Shochrul Rohmatul Ajija, Raditya Sukmana, Tita Novita Sari, Ahmad Hudaifah

**Affiliations:** a Department of Economics, Universitas Airlangga, Indonesia; b Department of Shariah Economics, Universitas Airlangga, Indonesia; c Department of Islamic Economics, Universitas Internasional Semen Indonesia, Indonesia

**Keywords:** Breadth and depth outreach, Islamic cooperatives, Size, Non-performing finance, Grant, Corporate finance, Financial economics, Behavioral economics, Social inequality, Social responsibility, Economics

## Abstract

This study aimed to calculate the breadth, depth, and overall outreach score with case studies of several Islamic cooperatives in East Java. Generally, the level of this outreach is not too high, but it continuously showed an increase from 2014 to 2018. Those cooperatives with relatively small assets tend to have high outreach scores. Meanwhile, those with large assets have a lower outreach level. Therefore, this study utilized the Tobit regression analysis in order to investigate the factors that influence outreach score. The results showed that size, non-performing financing (NPF), number of branches, grants, financial leverage, and age have a significant impact on Islamic cooperatives' outreach. An interesting finding is that size has a negative effect. This is in contrast with the spirit to develop cooperatives in Indonesia. Also, a high NPF can significantly decrease the level outreach. Meanwhile, the increase in the number of grants appears to have a positive impact. Thus, through action research since 2012 and FGD on the management of Islamic cooperatives, this study provided an explanation on why these conditions can occur.

## 1 Introduction

Poverty is known to be a severe social problem in Indonesia. According to a report by the Statistic Bureau (Badan Pusat Statistik, 2018), about 25.9 million people or approximately 9.82% of the entire population are living in penury. This situation and its adversity makes people and the community vulnerable to economic problems. It also makes it difficult to meet the regular standards of living, thereby affecting their welfare attainability (Jacobus et al., 2018).

A familiar indicator to measure and gauge the level of welfare and well-being is Human Development Index (HDI). This is a holistic and multi perspective parameter that enables policy makers to properly evaluate community development from an economic, health, and educational perspective (Mariyanti and Mahfudz, 2016). The United Nations Development stated that in 2018, Indonesia's HDI is ranked 116 out of 189 countries in the world. Therefore, poverty alleviation remains a top priority that the government needs to properly address.

One possible alternative to cope with this problem is to set up a reliable and continuous micro finance system through an institution and cooperative. Such approach will prompt and ignite a wider spectrum for societal empowerment, which will ultimately improve welfare as well as employment opportunities (Rofiah, 2010). According to act No 1 2013, Micro-finance Institution (MFI) is a financial industry created to develop micro-businesses and empower the community. This is achieved through various monetary services, such as savings management, consultancy, and business development. Hermes and Lensink (2011), defined it as an institution that focuses on providing credit to those without access to commercial banks, and have a business that generates profits. Through MFI, the society is empowered and economically nurtured to improve financial independence, with positive welfare intervention that creates beneficial impacts such as employment and job opportunities.

According to Act No.1 2013, the MFI contract scheme and service which aims at financing and saving is applicable to Islamic and non-islamic institutions. The Islamic Micro-finance Institutions (I-MFI) are entities that are managed using *Sharia* principles. In Indonesia, they operate and work in accordance with the fatwa issued by the National *Sharia* Council (DSN MUI). This institutions are expected and considered to be a pro-income redistribution for communities, and does not involve high interest rate for transactions. MFIs implementation and submission of interest based-system are considered to be against the concept of just income redistribution in the community. Therefore, Islamic institutions provide loans to the poor without repayment terms such as fixed interest at the beginning of the transaction. There is every possibility that the borrower does not always make profit, or even suffers losses. Hence, the profit-sharing principle superiority of Islamic cooperatives is considered fair, just, and beneficial to both parties. This is because it is based on business profits acquired from the circulation of money lent to the public (Susamto, 2012).

In addition, Islam strictly forbids usury, which is a form of interest on loans. This term means additional (*ziyadah*) or to grow (Saeed, 1996). In terminology, it is interpreted as a return from basic assets (Susamto, 2012). The National *Sharia* Council *Fatwa* Number 1 of 2004 stated that the current practice of interest transactions has fulfilled the criteria of usury, hence the law is prohibited by various institutions. Therefore, I-MFI is considered to be more prosperous to the community.

Islamic cooperative is a predominantly existing and emerging application of I-MFI in Indonesia, and is defined as people's economic movement based on the principle of kinship (Soemitra, 2018). It develops around the community due to character match that upholds the value of cooperation. In 2016 the number of active cooperatives in the country was 148,220 and the majority are in East Java (Badan Pusat Statistik, 2018).

Agbola et al. (2017) as well as Adnan and Ajija (2015) stated that Islamic cooperatives play an important role in poverty alleviation. Similar study and examination were also conducted by Gina and Effendi (2015) which stated that I-MFI financing had a positive effect on increasing the income of micro entrepreneurs, as well as having a substantial impact on people's welfare. Furthermore, Muljadi (2018) stated that their role in the local formal terminology is similar to *Baitul Maal Wat Tamwil* (BMT) which was strategic in reducing poverty.

The results of the above-mentioned study raised concerns on the number of poor people in East Java which remained high at the end of 2018. According to Badan Pusat Statistik (2018), this region has 94.58% Muslim population and comprises of about 4.1 million poor faction. In 2019, about 14.43% of the total residents lived in poverty. When compared to other provinces, it ranked 9^th^ in a sequential order of Papua, West Papua, Maluku, East Nusa Tenggara, Gorontalo, Aceh, Bengkulu, and Central Sulawesi.

The paradox of the large number of cooperatives and high poverty rates is certainly a serious concern. The increase in the number of poor people is certainly not in accordance with the objectives of the cooperatives, which is to prosper the community Zeller and Meyer (2002) as well as Adnan and Ajija (2015). Therefore, cooperatives need to be prepared with a relatively high default risk when they intend to deal with a poor population (Hermes et al., 2011).

Several previous studies have measured the affordability level of micro-finance institutions. However, the researches showed that majority of these firms have not financially impacted the livelihood of the poor. The studies (Henock, 2019) stated that most of these institutions had a moderate level on their outreach level. Wijesiri et al. (2017) showed that most of the older MFIs in Asia, Latin America, Africa, and Eastern Europe are inefficient in achieving their outreach objectives. There are other researches which include Mulyaningsih et al. (2017) at *Sharia* micro-finance institutions in Bogor, West Java; and Rifai et al. (2018) at the Savings and Loans Village Economic Business Micro-finance Institution in Rambah District, Rokan Hulu Regency.

Some studies indicate several cases of MFIs that were default in attaining their outreach but no research on measuring its level. Therefore, this study aimed to measure the cooperatives' outreach for the poor, and the factors that influence its affordability. Furthermore, it also provides sustainable solutions to I-MFIs in accordance with the initial purpose of microfinance, which is to improve the welfare of small communities. This is believed to contribute to the breakthrough of alleviating poverty in the country.

## 2 Literature review

Outreach on communities refers to the interactions and relationships of MFIs with both internal and external stakeholders, and it involves contributing to the welfare of the society. Generally, the outreach and accessibility of financial services is identified and gauged by the available institutions (Mulyaningsih et al., 2017). However, its related indicators and accessibility are measured by the number of clients served. This is done by ascertaining the average loan size and the percentage of borrowers, to measure the depth and length of reachable service. Also the relationship between cooperative and client, with installment and credit payment period is measured (Henock, 2019).

There are two subdivisions of range, which are depth and breadth. The Depth of coverage shows an increase in the number of community assisted with credit services. Meanwhile, the breadth of recommendations is served with micro credit (Handayani, 2013). Arsyad (2008:174) stated that the level and rate of outreach is determined by the average loan (savings or deposit) to borrower ratio, and the average financing per member. The coverage depth indicator reflects the value obtained by the community from the net assessment of certain participants.

Many distinctive approaches are used to determine the variables of measuring the depth and breadth of outreach. However, this study used those identified by (United States Agency for International Development (USAID), 2006), which consists of broad indicators such as the number of borrowers, percentage of non-productive activities, and number of voluntary savers. It also consists of clients' percentage using MFI services, and the total percentage accessible to non-financial facilities, such as training and other empowerment programs. Meanwhile, the depth indicator comprises of the average percentage of credit given to customers per Gross National Income (GNI), loans under $300 (equivalent to Rp. 4,027,500), number of village borrowers, and the percentage of loans to entrepreneurs targeted by poverty alleviation programs.

According to some literatures, the size of a cooperation has a positive impact on achieving better outreach performance (Henock, 2019). When institutions own more assets, they will have enough resources to address the needs of loan seekers. Also, size contributes positively to both the financial and social efficiency, suggesting that the larger the institution, the more its strength in terms of sustainability and poverty outreach (Wijesiri et al., 2017). This can be attributed to the ability of larger MFIs to reduce the costs from scale economy. Another possible explanation for this positive relationship is that the larger MFIs may use more sophisticated technologies (i.e. advanced management information systems, teller machines, online transactions, mobile banking) and their ability to diversify products and services (i.e. savings mobilization, remittance, insurance, or leasing) through a well-established network of branches. This helps to improve financial inclusion in an effective way, compared to smaller institutions that depend on outdated methods. Therefore, the hypothesis was proposed as follows:

H1 *Cooperative size has a positive effect on outreach*

The higher donation amount has a positive influence on the depth of outreach because it generates more loanable funds (Henock, 2019). Based on the description, the hypothesis proposed is as follows:

H2 *Grants positively influence outreach*

Nkundabanyanga et al. (2015) stated that outreach performance of the MFIs depends on the manager's competence in calculating risks. A reduced risk will minimize the costs incurred, therefore providing adequate financial support to the poor**.** Based on the description, the hypothesis proposed is as follows:

H3 *The employee education level has a positive effect on outreach*

Henock (2019) stated that there is a positive relationship between debt equity ratio and outreach performance. This is because cooperatives can mitigate credit market failure by providing financial services tailored to the poor and low-income earners. When the asset, debt, and profitability of the institutions increase, more funds will be generated and used to significantly increase outreach depth. Based on the description, the hypothesis proposed is as follows:

H4 *Leverage has a negative effect on outreach*

Wijesiri (2017) stated that the older and larger MFIs are more likely to get funds from their members than smaller institutions. Since they have more money, then there is higher ability to perform better. Rifai et al. (2018) also stated that older institutions give more credit loans to customers. Based on the description, the hypothesis proposed is as follows:

H5 *Age has a positive influence outreach*

According to Handayani (2013), financing to deposit ratio (FDR) has a positive impact on MFIs ability to increase their credit disbursed to customers. Accordingly, the probability to improve their outreach also increases. Based on the description, the hypothesis proposed is as follows:

H6 *The intermediation function or FDR has a positive effect on outreach*

Handayani (2013) stated that the number of offices is negatively related to outreach. This is because the locations of the central offices and banks are in the middle of the city which is usually dominated by high income individuals. Therefore, clients who reach them are not in accordance with the objectives of poverty outreach. Based on the description, the hypothesis proposed is as follows:

H7 *The number of offices negatively influences the outreach*

Using a case study on rural banks (BPR), Rifai et al. (2018) found that the number of customers from the agricultural sector had a negative effect on outreach. This was because those who could access the BPR funds were small and medium scale farmers. This was indicated because they already had collateral that could be guaranteed to get funding. Therefore, the hypothesis proposed is as follows:

H8 *Agricultural sector customers negatively affect outreach*

Handayani (2013) stated that return on assets (ROA) has a negative effect on achieving outreach depth. When an institution serves the poor, the costs incurred will be greater. Therefore, the financial institutions that pay attention to depth of outreach are more likely to have small operating profits with low return on assets. Based on the description, when the MFIs set a higher ROA target, then the outreach becomes lower. Accordingly, the hypothesis proposed is as follows:

H9 *Targeted ROA has a negative effect on outreach*

In this study, the Non-Performing Financing (NPF) ratio was appended as a variable that affects the range and grants received by Islamic cooperatives. When the ratio increases, profitability decreases, leading to reduced channeled funds (Wibowo and Syaichu, 2013). Similarly, according to a study conducted by Widayatsari (2013), an increase in NPF decreases the disbursed finances. From this explanation, the NPF is assumed to have an effect on outreach. However, due to the unskilled nature of poor people and their inability to properly manage funds, they are vulnerable to unnecessary exposure. Based on the description, the hypothesis proposed is as follows:

H10 *NPF has a negative effect on outreach*

## 3 Data and research method

This research used qualitative methods to measure the outreach level of Islamic cooperatives in East Java. Outreach is the ability of MFIs to provide and supply financial services to many clients. In this study, it is focused on two categories, which are depth and breath as explained and analyzed by Schreiner (2002). The breadth measures the number of clients that are able to acquire credits from the MFI, while the depth measures the number of assisted poor people.

Quayes (2012) stated that variables need to be determined to quantify the depth and breadth of outreach. In addition, the information regarding the total assets and income levels of each customer need to be accurately obtained for proper analysis. However, there are many limitations in obtaining such information. Therefore, the proxy employed to measure deeper outreach is the average amount of credit received by customers (Awaworyi and Marr, 2014). This is because there is a positive relationship between income level and the amount of credit received by clients (Cull et al., 2007; Quayes, 2012; Ameer, 2016). In addition, the number of female borrowers is also a proxy for measuring outreach due to their vulnerability to poverty (Bhatt and Tang, 2001). Therefore, the measurement is conducted by calculating the number of active borrowers at the head, branch, and cash offices (Awaworyi and Marr, 2014; Ameer, 2016) (see Table 1).

**Table 1:** Indicators of outreach index.

Outreach	Indicator		Scale	Data Source
Breadth	1	The Number of Financing Receiver	0 → < 20.000 1 → 20.000–50.000 2 → > 50.000	Islamic cooperative financial statements
	2	The percentage of financial aid recipients used for non-productive activities	0 → < 10% 1 → 10%–30% 2 → > 30%	Interviews and financial statements of Islamic cooperatives
	3	The percentage of voluntary savers compared to total fund recipients	0 → < 50% 1 → 50%–75% 2 → > 75%	Interviews and financial statements of Islamic cooperatives
	4	The Percentage of members that use MFI services such as transfers or insurance to the total recipient of the fund	0 → < 10% 1 → 10%–30% 2 → > 30%	Interviews and financial statements of Islamic cooperatives
	5	The percentage of members that received non-financial facilities such as training or other empowerment programs to the total recipient of the fund	0 → < 10% 1 → 10%–30% 2 → > 30%	Interviews and financial statements of Islamic cooperatives
Depth	1	The average percentage of funding provided to members per Gross Domestic Regional Product per capita in East Java	0 → > 100% 1 → 60%–100% 2 → < 60%	Financial reports of Islamic cooperatives and the Central Statistics Agency
	2	The Percentage of financing under $300 (equivalent to Rp.4,027,500)	0 → < 20% 1 → 20%–50% 2 → > 50%	Interviews and financial statements of Islamic cooperatives
	3	The percentage of female financing recipients	0 → < 20% 1 → 20%–50% 2 → > 50%	Interviews and financial statements of Islamic cooperatives
	4	The percentage of recipients from villages	0 → < 15% 1 → 15%–30% 2 → > 30%	Interviews and financial statements of Islamic cooperatives
	5	The percentage of financing to entrepreneurs originating from poor customers targeted by the poverty alleviation programs	0 → < 20% 1 → 20%–50% 2 → > 50%	Interviews and financial statements of Islamic cooperatives
Total Maximum Score			20	

Source: USAID (2006).

One of the difficulties in collecting data on points number 2 to 5 on the breadth and depth outreach indicators was that the database of the cooperative members was still not organized. Therefore, answers to these points were directly obtained from interviews with the directors and managers of cooperatives. In addition, outreach index from each MFI was calculated based on the following formula:

(1) $$\;\;Outreach\;Index=\frac{Total\;Score\;of\;Outreach_{i}}{20}\times 100$$

In order to determine the factors that influence outreach index, the Tobit regression model was used on the panel data collected from 8 Islamic cooperatives from 2014 to 2018. The samples were members of the East Java *Sharia* Cooperative Forum (FKS). Out of the 95, there were only 16 members willing to be investigated. In fact, some were not pleased to be researched because they thought it is irrelevant to their business process. They argued that the problem of poverty is more appropriate when it is linked to the *Baitul Maal* program than the *Baitul Tamwil*. This is because these cooperatives have a great responsibility in managing the *ummah* funds for it to be appropriately channeled to people. Furthermore, from the 16 Islamic cooperatives, only 8 have complete data and can be further analyzed (see Table 2).

**Table 2:** Lists of Islamic cooperatives in East Java calculated for outreach level.

Name of Islamic Cooperatives	Existence Period (Years)	Asset∗ (IDR)	Groups
BMT Muda	6	2.479.323.763	1
BMT AL Izzah	8	3.108.845.661	
KSPPS MUI	7	25.212.069.977	2
BMT Permata	9	12.219.768.382	
Mandiri Artha Syariah	11	1.492.199.227	3
ArthaInsani Banyuwangi	21	7.793.081.137	
Kanindo Syariah	21	44.459.115.827	4
KSPPS BMT DMU	23	31.766.730.355	

Note: ∗Per December 2018.

To determine the factors that influence outreach index, the authors use the Tobit panel model. The equation for the outreach index determinant is as follows:

(2) $$OI_{it}=\alpha_{0}+\alpha_{1}size_{it}+\alpha_{2}finlev_{it}+\alpha_{3}grant_{it}+\alpha_{4}educ_{it}+\alpha_{5}npf_{it}+\alpha_{6}roa_{it}+\alpha_{7}fdr_{it}+\alpha_{8}branch_{it}+\alpha_{9}farm_{it}+\alpha_{10}age_{it}+\mu_{it}$$

More specifically, the variables used in this model are as follows (see Table 3):

**Table 3:** The definition of variables.

Variable	Definition
OI	Outreach Index
Size	Natural logarithm of total assets
finlev	Financial leverage is measured by the ratio of the total sources of all funds used by Islamic cooperatives in addition to capital
Grant	Ratio of total grants to equity
Educ	Percentage of employees with a minimum of bachelor's degree
NPF	Non-Performing Financing
ROA	Return on Asset
FDR	Finance to deposit ratio
Branch	Number of branch offices, cash offices, or service counters owned
Farm	Proportion of customers from the agricultural sector
Age	Age of Islamic cooperative

According to Law No. 25 of 1992 article 41, cooperative capital (the right side of the balance sheet) is obtained from an individual's loans. Such asset and liability are generated from principal, mandatory, reserve funds and grants. The principal deposits are the amount of funds and contributions paid by all members at the beginning of registration. While, mandatory deposits are shares paid per month. The reserve funds are obtained from the profits of previous year which are reserved as capital. The loan capital is supported from internal members, cooperatives, banks, and other financial institutions, with the issuance of bonds and debt securities from legal sources. In the context of Islamic cooperatives, loan often originates from a variety of funds received from other financial institutions. However, in this study, leverage calculation does not include grant funds in the private capital components. This is due to the need to determine the influence of Islamic grants, with respect to the government and private sectors.

This study used Tobit regression because the dependent variable consists of censored data. The number 0 on the variable shows Islamic cooperatives are unable to facilitate the poor, while a score more than 0 to a maximum of 100 indicates its ability to connect and assist access to finance. Greene (2008) defined censored data as the number that is limited to a certain range. Therefore, the regression was used on mixed data to reduce and diminish the effects of bias when compared to the data processed using a classical linear regression. This is because censored and continuous data are created, therefore information is not lost. The Tobit method assumes that free variables are limited, censored, and independent without using autocorrelation, heteroscedascity, perfect multicollinearity, and mathematical model (Gujarati, 1995).

Furthermore, to confirm the findings through a quantitative approach, a focus group discussion (FGD) was conducted. This activity was attended by 32 management representatives and managers from 16 cooperatives in the East Java. The analysis results were confirmed by action research through the establishment of Islamic cooperatives since 2012. By December 2018, its members had grew to 582 with assets worth 2.5 billion Rupiah. Therefore, it is expected that the research analysis develops a more comprehensive information and insight on the processes and management constraints of Islamic cooperatives.

## 4 Finding and discussion

### 4.1 Outreach index calculation

The calculation of outreach index was based on breadth and depth of the developed indicator (USAID, 2006). Overall, the profiles of the 10 indicators obtained from interviews and data documentation from 8 Islamic cooperatives from 2014 to 2018 are as follows (see Table 4):

**Table 4:** Score of each components of outreach index.

Variable	Obs.	Mean	Std. Dev.	Min	Max
Number of finance recipients	40	3103	7665	15	33504
The percentage of finance recipients used for non-productive activities	40	26.39	24.67	0.1	96.70
The percentage of voluntary savers compared to the total finance recipients	40	187.32	142.71	101	1000
The percentage of members that use MFI services such as transfers or insurance to the total finance recipients	40	48.98	37.04	0	90
The percentage of members with non-financial facilities such as training or other empowerment programs to the total funding recipient	40	13.07	13.96	0	50
The average percentage of funding provided to members per Gross Domestic Regional Product per capita in East Java	40	52.95	62.58	1	207
The percentage of financing under $ 300 (equivalent to Rp.4,027,500)	40	38.93	27.85	5	90
The percentage of number of female financing recipients	40	49.35	25.88	9	90
The percentage of recipients of funding from villages	40	65.60	28.81	5	100
The percentage of financing to entrepreneurs originating from poor customers targeted by the poverty alleviation programs	40	26.13	30.97	0	100

From these indicators, outreach indexes were calculated based on the (USAID, 2006) method. Generally, the average index for the 8 cooperatives is as follows (see Table 5):

**Table 5:** The outreach index of respondents from 2014 to 2018.

Outreach	2014	2015	2016	2017	2018
Breadth	0.44	0.45	0.54	0.53	0.56
Depth	0.60	0.61	0.61	0.61	0.60
Overall	0.52	0. 53	0.57	0.57	0.59

The cooperatives with the highest overall outreach score among 8 others are BMT Mandiri Artha Syariah and BMT Al-Izzah, which financed and channeled a number of funds. BMT Mandiri Artha Syariah can improve the income of poor people in Bojonegoro region by providing business training and capital. These efforts are effective and tend to increase income.

Meanwhile, BMT Al-Izzah aims to reduce and alleviate poverty from communities around its business location. This is done by releasing the people from interest-based debt from loan sharks, pawn shop, and usurious system. Many communities around BMT Al-Izzah earn and make little incomes for a living, and even meet the needs to pay off their debts. Therefore, this institution provides non-interest loans to people trapped by moneylenders, in accordance with Islamic agreements.

Both of these cooperatives have succeeded in fulfilling and meeting the characteristics of corporate social responsibility based on Islamic perspectives, one of which is reducing poverty. In Islam, prosperity is not only meant for the rich, but also for poor communities as stated in *Quran* surah Al-Hasyr verse 7. In addition, BMT Al-Izzah Amanah Umat has succeeded in helping the people trapped by usury, which is a great sin and guilt in Islam.

The outreach index score also showed that most respondents achieve higher depth values, with an average below 0.50. This means that majority are still unable to reach the wider community. Also, there are Islamic cooperatives that generate a score of 0.0 in terms of breadth outreach and majority of them are able to channel finance to more than 20,000 members as required in the MFI outreach calculation. In addition, respondents that channel non-productive finance on spot needs, education, and health is low. With the existence of non-productive financing, it is expected that members can help meet the basic needs of the community to improve their productivity and standards of living.

Unattainable optimal outreach breadth of respondents is also caused by insignificant 3 indicators in the equation and model. In the first, there is less dominant percentage of voluntary savers compared to the total recipients with limited low-cost and flexible internal source of income. Secondly, it is influenced by the low number of members using the cooperative services such as transfers and insurance. This is because the financial services are not extensive compared to banks. As a result, the range of services provided to the wider community is also limited. Thirdly, the minimum percentage of members that received non-financial programs and the total recipients of funding also worsened the insignificant score and indicator.

Although the average breadth outreach remains inadequate, its cumulative score is above 0.50. This means the respondent is able to reach the poor. The indicators that considerably contribute to higher outreach depth are the members from villages, the average percentage of funds, and the number of female recipients. Meanwhile, there are lacking indicators which has limited respondents' outreach with nominal (micro) funding.

The lower levels of the two depth outreach indicators are an interesting finding in the field of study. Islamic cooperatives as representatives of microfinance institutions need to be prompted as a medium to reduce poverty. This can be done by providing access to finance for those who are unable to obtain loans from commercial banks (Hermes et al., 2011). Some respondents claimed it was difficult to embrace and engage the poor in Islamic microfinance tract. Less favorable poor communities are left behind due to excessive risk, and in most cases, financing becomes problematic. This is because funds received from cooperatives are utilized to fulfill basic needs, without adequate source of income. For this reason, most respondents ultimately prefer to channel funds to established-small businesses rather than those managed by the poor. Although respondent's depth of outreach is quite good, but its breadth is low. This in-turn leads to sub-optimal average overall outreach. Generally, most cooperatives that achieve an outreach above 0.50 are those in cluster 1, and are less than 10 years old with assets under 10 billion Rupiah (see Table 6).

**Table 6:** The outreach index for each Islamic cooperative.

Name of Islamic Cooperatives	Group	Breadth	Depth	Overall
BMT Al Izzah	1	0.68	0.94	0.82
Mandiri Artha Syariah	3	0.48	0.94	0.71
BMT Muda	1	0.62	0.64	0.63
BMT Permata	2	0.7	0.52	0.61
Artha Insani Banyuwangi	3	0.5	0.46	0.48
Kanindo Syariah	4	0.26	0.56	0.41
BMT DMU	4	0.42	0.38	0.40
KSPPS Mitra Usaha Ideal	2	0.36	0.42	0.39
**Average**		**0.50**	**0.61**	**0.56**

Large scale and long-standing cooperatives tend to have low outreach levels due to their experience in dealing with small communities with high risk of sustainability. Generally, large cooperatives are no longer focused on assisting the poor because of their high risks in financial activities. Therefore, this goal has been delivered to an amil *zakah* institution, which is independent with separate finances. Hence, Islamic cooperatives are focused on productive businesses and generating profits, whereas the amil *zakah* Institute is aimed at empowering the poor.

### 4.2 Determinant of outreach index

After calculating the outreach index score, the factors that influence it was identified. There are ten variables that presumably impact the index. They are shown in the following table (see Table 7):

**Table 7:** Descriptive statistics of factors affecting outreach index.

Variable	Obs.	Mean	Std. Dev.	Min.	Max.
size	40	22.64	1.07	21.12	24.52
age	40	11.28	6.90	2	23
finlev	40	586.48	310.21	152.35	1,396.60
educ	40	45.25	31.01	-	100.00
npf	40	3.46	2.58	-	9.16
grant	40	6.93	9.07	-	18.83
roa	40	2.88	2.66	0.15	11.32
fdr	40	100.54	45.48	15.09	189.27
branch	40	4.43	4.24	1	14
farm	40	20.00	9.42	10	43

From the table, age is measured from the year of establishment. Furthermore, the data indicated that KSPPS DMU is the oldest Islamic cooperative with 23 years in service, while BMT MUDA was 2 years in 2014. In addition, the financial leverage of respondents is tremendously high with about 1,396.60. This means they are highly dependent on external funding sources, such as member's savings, banks, and other association. In terms of service period, the most senior and experienced respondent was KSPPS BMT DMU and the youngest entity was BMT MUDA.

On average, less than half of the respondents' employees have a bachelor's degree in education. Since 2017, all KSPP Mitra Usaha Ideal employees are managed by staff and team with bachelor's degree. In contrast, none of the Artha Insani Banyuwangi cooperative employees have a degree in education background.

The average NPF level of respondents is quite low. This standard of classification varies between banks and cooperatives. This is because most of the members are micro-businesses that have a higher risk. According to the Bank of Indonesia, there are five categories in the NPF level, which are: Rating 1 = NPF <2%, 2 = 2% ≤ NPF <5%, 3 = 5% ≤ NPF <8%, 4 = 8% ≤ NPF <12%, and 5 NPF ≥12%. Meanwhile, based on Deputy Supervision Regulation No. 06/Per/Dep.6/IV/2016 and No. 07/Per/Dep.6/IV/2016 concerning guidelines for the evaluation of savings and loan cooperatives, the ratio of problem loans to cooperatives that get the best assessment score is less than 10% (Ajija et al., 2018). Thus, NPF becomes an important variable in the outreach analysis. This is inseparable from the activities of Islamic cooperative, which is associated with micro-small businesses that are prone to default risk. During the analysis, there was a high NPF of 9.16 in KSPPS Mitra Usaha and 9.1 in BMT MUDA in 2014. Thus, it can be concluded that the cooperatives sampled in this study have good loan performance.

This research also strived to split financial grants in the capital component, with the aim of determining its effect on outreach performance in the country. In addition, only three respondents in BMT Permata and Kanindo Syariah where each funded with 150 million Rupiah and KSPPS BMT DMU with 55 million.

According to Dwi (2012), ROA is a ratio used to quantify a company's ability to generate profits using its total assets. Therefore, the higher the ROA, the more the level of profitability. Based on the data obtained, the average respondents ROA remains limited at 2.88%. The smallest return was obtained by BMT Mandiri Artha Syariah, which was 0.15% in 2014. Meanwhile, BMT Al-Izzah, obtained the highest at 11.32% in 2017.

Financing to Deposit Ratio (FDR) is the comparison between the funds provided by banks and a third party (Widyaningrum and Septiarini, 2015). An increase in the FDR ratio shows that there is a rise in monetary distribution to the public. Therefore, when this ratio rises, bank profits also increase with the assumption that its finance was optimally channeled. The highest FDR was achieved by BMT MUDA in 2016 and the lowest by BMT Permata in 2018.

Also, the number of offices showed the total branch and network facility owned by Islamic cooperatives. Hence, an increase in this number has the ability to make the transaction easier for the people. The average number of offices owned by Islamic cooperatives is approximately 4 units agencies and workshops. Those within the study area are predominantly owned by Kanindo syariah. Meanwhile, the cooperative with the least number of offices comprises of BMT Artha insani and BMT Al-Izzah.

Recipients from the agricultural sector are considered to influence outreach. This is because the aspect is generally managed by poor people living in rural communities (Rifai et al., 2018). The average cooperative member in the sector is around 20%. Meanwhile, the largest number of recipients comes from BMT DMU, and the less members are in Kanindo Syariah.

The Tobit panel regression model was obtained with the factors that influence outreach using the estimation results. These are shown in the following table (see Table 8):

**Table 8:** Output of Tobit regression.

Variables	(1)	(2)	(3)	(4)
cons	5.082	2.070	2.138	3.345
	(0.543)∗∗∗	(0.848)∗∗	(0.011)∗∗∗	(0.772)∗∗∗
size	-0.200	-0.816	-0.846	-0.126
	(0.0262)∗∗∗	(0.035)∗∗	(0.034)∗∗	(0.034)∗∗∗
finlev	-0.000	-0.000	-0.000	-0.000
	(0.000)∗	(0.000)	(0.000)	(0.000)
educ	0.000	0.000	0.000	0.000
	(0.009)	(0.000)	(0.001)	(0.000)
npf	-0.030	0.011	0.011	-0.014
	(0.007)∗∗∗	(0.008)	(0.008)	(0.008)∗
roa	0.006	0.000	0.000	0.004
	(0.007)	(0.008)	(0.008)	(0.004)
fdr	-0.000	-0.000	-0.000	0.000
	(0.000)	(0.000)	(0.000)	(0.000)
branch	0.020	0.014	0.153	0.171
	(0.005)∗∗∗	(0.009)	(0.008)	(0.007)∗∗
age	0.000	0.0217	0.022	
	(0.005)	(0.009)∗∗	(0.008)	
farm	-0.001	-0.000		
	(0.001)	(0.002)		
grant	0.009			
	(0.002)∗∗∗			
Wald Chi Square	181.56	24.17	24.32	17.29

Note: ∗,∗∗, and ∗∗∗ are significant at 10%, 5%, and 1% respectively.

The numbers inside the brackets indicate the standard deviation.

From the estimation results, it was concluded that 3 variables have different results with the hypothesis, i.e size and branch. The size of cooperatives consistently exhibits a negative effect on overall outreach. Furthermore, the results of this regression confirm the outreach calculation which showed that the cooperatives in group 4, were established earlier with large assets and low outreach. Therefore, the results contradict and disagree with previous research such as (Henock, 2019) and Wijesiri et al. (2017), which stated that the size of MFI has a positive effect on outreach.

Based on FGD results, the discussion found that large cooperatives differentiate interest to help the poor achieve commercial goals. These institutions prefer to construct and manage their own *Baitul Maal* (house of philanthropy and almsgiving) which maintain a separate and independent financial report. Also, it focuses on empowering fragile, vulnerable, and poor communities. This is because such initiative examines and presume from a business perspective. Therefore, these conditions need to be minimized in the development of an Islamic cooperative. However, due to their initial goal to improve the people's welfare, they set up their own institutions to focus on developing businesses from these communities.

The negative effect of size on outreach raises a big question considering that it is one indicator of success in cooperative management. When deeply examined, grants have a positive effect, but leverages have a negative impact on outreach. Therefore, it needs to be more detailed in analyzing the source of funds. This is also confirmed by the signal that negative FDR coefficient is also one indicator for deeper investigation of cooperatives' assets. It was confirmed from the FGD results that when cooperative funding sources are dominated by external funds such as banking or other channeling programs, then the institution will focus on disbursing the funds to members who have feasible businesses. Meanwhile, the poor could be a problem because they often delay in installments. Therefore, the cooperative directs the poor who want to get access to funding through *Baitul Maal*. After they have been successfully fostered, then it is likely they will become partners.

The number of branch offices does not consistently have a significant effect on the outreach level. However, an increase in number tends to encourage the ability of cooperatives to serve wider and broader society. Unlike Handayani (2013) which stated that BPRS branch offices have a negative effect on outreach, this study showed that more branches improve outreach. This is because the branch offices are closer to their members in remote villages. One of the challenges faced by Islamic financial institutions is the low level of public literacy regarding products and services (Akmal and Saputra, 2016). Therefore, the presence of cooperative offices in the community makes it easier to reach out to the poor, thereby increasing their opportunity to access funds. However, the results of this study are not in accordance with those conducted by (Handayani, 2013), which stated that the number of offices has a negative and significant effect on outreach depth with the assumption that other variables remain constant. This is because the location of most head and branch offices are in the middle of the city which is densely dominated by high income individuals. Therefore, the results of this study showed the positive influence of high number of offices.

Furthermore, the hypothesis that the older the cooperatives, the more their outreach was confirmed. This is because the longer they run a business in the cooperative, the more they will understand the character of each member.

NPF is a variable that has a significant negative effect on outreach, with the ability to reduce profitability and confidence of third-party investors. Therefore, cooperatives have become thorough in channelling funds to avoid risky financing. The poor are denied access to funds because they are perceived as helpless with no specific skills in starting up, managing, and scaling up a business project. Nevertheless, KSPPS ideal business partners who have the highest NPF have a lower outreach score compared to BMT MUDA. This certainly needs further analysis, whether the cause of the high NPF is due to the mistakes of micro-business members, or because the internal management is poorly organized.

Meanwhile, FDR has a positive effect on outreach, although the multiplier effect is close to zero. When Islamic cooperatives link more funds, then the possibility to reach a wider community including the poor is also higher. Furthermore, various cooperatives receive funds and linkage from other financial institutions which are also considerably high with significant asset contribution. This is done to maintain liquidity while increasing the volume of cooperative businesses in order to provide services to the wider community.

In line with the research conducted by (Henock, 2019), a notable finding in this study is that grants have a positive effect on outreach. These funds are initiated and supported by the government and corporate social responsibility (CSR) source from private and business entities. According to Huda et al. (2017), a grant is a voluntary gift in the form of assets from one party to another. Therefore, the higher the amount received; the more funds distributed.

## 5 Conclusion

This study aimed to analyze the outreach of Islamic cooperatives towards the poor in East Java, as well as the factors that influence them. Based on the presented analysis, it was concluded that those with assets under 10 billion Rupiah have better outreach. Conversely, large-scale cooperatives seem to exhibit a lower and insignificant level of outreach. More specifically, BMT Mandiri Artha Syariah and BMT Al Izzah had the highest overall score among the 8 samples. BMT Mandiri Artha Syariah is an Islamic cooperative categorized in group 3, but its assets remain continuously small. Meanwhile, Al Izzah BMT is placed in group 1 that is comparably young with little assets.

In addition, when comparing the components that make up the overall outreach, it was ascertained that the average cooperative has a higher depth. This is because the three indicators forming the depth outreach have high scores on the number of members coming from villages, the average percentage of disbursed funds, and female beneficiaries. Furthermore, other indicators remained statistically insignificant and does not affect micro and small nominal funding services channeled to the poor.

The slight and inconsiderable outreach score in larger and more established cooperatives is due to their role in empowering the poor. Therefore, an outreach analysis on large-scale cooperatives needs to include the Baitul Maal Institute. Furthermore, the synergy between Baitul Maal and these institutions needs to be improved, especially in channeling productive financing to the poor.

In accordance to the breadth outreach, three factors are responsible for the low score. An example of such variable is the limited affordable source of funds. Therefore, the role of cooperatives is not optimal in payment services and transfers, or insurance. Furthermore, from the Tobit regression estimation results, it was concluded that the overall outreach is influenced by the size, NPF, branch, grant, financial leverage, and age. The higher the level of assets, NPF, and financial leverage, the lower the overall outreach and vice versa. In contrast, grant, age, and number of offices turned out to play a positive role.

To increase the outreach cooperatives, agencies need to channel and provide a reliable source of funds from the government. In addition, to enhance their role in providing non-financial facilities, the interested entities need to work together with external parties, such as universities, government, non-government organization, as well as private and state-owned companies. Also, efforts need to be made to suppress NPF as an inhibiting factor, which will tackle problematic financing.

Overall, this research stated the conditions and determinants of outreach in East Java. Its primary limitation is the examination of 8 Islamic cooperatives. Therefore, further analysis could not present its relative conclusive condition. Nevertheless, this study provided adequate contribution to cooperative literature since there was no research on this area.

## Declarations

### Author contribution statement

Wasiaturrahma and S.R. Ajija: Conceived and designed the experiments; Performed the experiments; Analyzed and interpreted the data; Contributed reagents, materials, analysis tools or data; Wrote the paper.

R. Sukmana: Performed the experiments; Contributed reagents, materials, analysis tools or data; Wrote the paper.

T.N. Sari: Performed the experiments; Wrote the paper.

A. Hudaifah: Contributed reagents, materials, analysis tools or data; Wrote the paper.

### Funding statement

The authors received funding from Kemenristek DIKTI, The Ministry of Higher Education of Indonesia.

### Competing interest statement

The authors declare no conflict of interest.

### Additional information

No additional information is available for this paper.

## Appendix A Supplementary data

Supplement Files Wasiaturrahma
